# Prognostic implications of sex-related disparities in severity assessment of primary mitral regurgitation following intervention

**DOI:** 10.1136/openhrt-2026-004573

**Published:** 2026-10-12

**Authors:** Edouard Long, Vitaliy Androshchuk, Hirra Yasir, Tiffany Patterson, Simon Redwood, Bernard Prendergast, Ronak Rajani

**Affiliations:** 1 Faculty of Life Science and Medicine, King’s College London, London, UK; 2 King’s College London, London, UK; 3 School of Cardiovascular Medicine & Sciences, King’s College London, London, UK; 4 Cardiovascular Directorate, St Thomas’ Hospital, London, UK; 5 Cleveland Clinic London, London, UK

**Keywords:** Echocardiography, Cardiac Surgical Procedures, Heart Valve Prosthesis, Mitral Valve Insufficiency, Women

## Abstract

**Background:**

Current guidelines advocate for intervention in primary mitral regurgitation (PMR) based on detection of severe MR using standardised thresholds. We sought to quantify sex-related differences in transthoracic echocardiography (TTE)-derived MR severity and long-term mortality after PMR intervention.

**Methods:**

Consecutive patients undergoing PMR intervention with validated vena contracta (VC) measurements were included. Proximal isovelocity surface area radius (PISAr), effective regurgitant orifice area (EROA) and regurgitant volume (RVol) were also measured in a subset of patients. Using Cox models adjusted for PMR mortality risk factors, VC was modelled as a restricted cubic spline with an interaction term for sex. Sex-stratified receiver operating characteristic (ROC) curves were used to identify potential differences in risk calibration.

**Results:**

Within 420 patients, females (n=168, 40%) displayed significantly lower absolute values of VC (median (interquartile range): 0.60 (0.55–0.80) vs 0.70 (0.60–0.80) cm, p=0.003), PISAr (0.85 (0.70–1.00) vs 1.10 (0.90–1.30) cm, n=96, p<0.001), EROA (0.35 (0.22–0.50) vs 0.60 (0.40–0.80) cm^2^, n=58, p=0.018) and RVol (51 (43–83) vs 88 (67–108) mL, n=53, p=0.013) compared with males. In multivariable regression, sex-related differences in long-term all-cause mortality were moderated by VC (P_interaction_=0.013). In ROC curve analysis, VC had similar discriminatory ability in both sexes (p=0.768), although the optimum cut-off was lower in females (females: 0.67 cm vs males: 0.74 cm).

**Conclusions:**

The present study demonstrates significant sex differences in TTE-measured MR severity and subsequent prognosis following intervention for PMR, which may merit sex-specific individualised thresholds.

**WHAT IS ALREADY KNOWN ON THIS TOPIC**

- Prior studies have shown that compared with males, female patients with primary mitral regurgitation (PMR) are less likely to undergo intervention and have worse long-term outcomes. Given evidence that females demonstrate significantly lower MR severity, with features of more advanced valvular disease, it follows that waiting for females to reach the same degree of MR severity as their male counterparts before offering intervention may inadvertently perpetuate suboptimal outcomes.

**WHAT THIS STUDY ADDS**

- In this study, we found that MR severity, quantified by transthoracic echocardiography-derived vena contracta, was a significant moderator of sex-related differences in all-cause mortality after PMR intervention. In addition, we identified sex-specific optimum thresholds for predicting mortality, with a lower cut-off in females.

**HOW THIS STUDY MIGHT AFFECT RESEARCH, PRACTICE OR POLICY**

- These findings suggest that sex-specific assessment of MR severity with individualised thresholds may facilitate more optimal timing of intervention in PMR and help bridge the gap in long-term outcomes between sexes.

## Introduction

 Over the past decade, it has become apparent that a ‘one-size-fits-all’ approach to primary mitral regurgitation (PMR) thresholds for intervention may be biased against females, and that they have worse long-term outcomes after intervention compared with their male counterparts.^1 2^ Current guidelines advocate intervention in PMR based on detection of ‘severe’ MR using thresholds applied equally to both sexes.^3 4^ Given the evidence that females demonstrate significantly lower MR severity compared with males, with features of more advanced valvular disease,^5^ it follows that waiting for females to reach the same degree of MR severity as their male counterparts before offering intervention may lead to suboptimal outcomes. Nevertheless, whether these disparities in MR severity have long-term prognostic significance remains unknown.

## Methods

Using the APEX registry of >8500 consecutive patients diagnosed with MR between June 2010 and November 2023, we identified those who underwent intervention for significant PMR and retrieved transthoracic echocardiography (TTE) performed within 6 months before the procedure. TTEs were performed by accredited operators and patients had validated vena contracta (VC) measurements, defined on colour flow Doppler imaging as the narrowest region of the MR regurgitant jet as it flows into the left atrium (LA). VC was chosen a priori as it is considered the most common and reproducible measure of MR severity in clinical practice. Proximal isovelocity surface area radius (PISAr), effective regurgitant orifice area (EROA) and regurgitant volume (RVol) were measured in a subset of patients.

Continuous variables are presented as median (IQR). Using Cox models, VC was modelled as a restricted cubic spline with three knots and non-linearity was assessed using the Wald test. The reference value was set at 0.7 cm, the guideline threshold for severe MR.^3 4^ Models were adjusted using established risk factors for PMR mortality (age, atrial fibrillation (AF), LA volume index, left ventricular (LV) end-systolic diameter index, LV ejection fraction (LVEF), tricuspid regurgitation (TR) grade and pulmonary artery systolic pressure (PASP)).^6^ Sex-stratified receiver operating characteristic (ROC) curve analysis was used to identify potential sex differences in risk calibration. The optimum threshold was defined as the Youden index, and the predictive power of variables was compared between sexes using DeLong’s test.

## Results

Among 420 study patients (table 1), females formed the minority (n=168, 40%) and were significantly older than males (74 (62–79) vs 70 (62–76) years, p=0.029), with no significant differences in rates of coronary artery disease, chronic obstructive pulmonary disease, prior cerebrovascular events, diabetes, hypertension, prior myocardial infarction or AF. There were no significant differences in rates of concomitant tricuspid (p=0.103) or aortic (p=0.214) interventions, nor in the type of intervention performed (MV repair/MV replacement/transcatheter MV repair: 106 (63%)/54 (32%)/8 (4.8%) vs 162 (64%)/77 (31%)/13 (5.2%), p=0.934). Females underwent less coronary revascularisation with bypass grafting (34 (20%) vs 77 (31%), p=0.019).

**Table 1:** Baseline clinical characteristics according to sex

Variable	Overall (n=420)	Female (n=168)	Male (n=252)	P value
Clinical variables				
Age, years	72 (62–77)	74 (62–79)	70 (62–76)	**0.029**
BMI, kg/m^2^	25.9 (23.2–29.3)	25.3 (22.3–29.3)	26.1 (23.6–29.2)	0.227
Coronary artery disease	72 (17%)	25 (15%)	47 (19%)	0.315
COPD	21 (5.0%)	10 (6.0%)	11 (4.4%)	0.465
Prior CVA	2 (0.5%)	2 (1.2%)	0 (0%)	0.159
Diabetes	31 (7.4%)	14 (8.3%)	17 (6.7%)	0.542
Hypertension	107 (25%)	48 (29%)	59 (23%)	0.235
Prior MI	3 (0.7%)	0 (0%)	3 (1.2%)	0.278
AF	71 (17%)	35 (21%)	36 (14%)	0.079
Concomitant TVr	79 (19%)	38 (23%)	41 (16%)	0.103
Concomitant SAVR	34 (8.1%)	17 (10%)	17 (6.7%)	0.214
Concomitant CABG	111 (26%)	34 (20%)	77 (31%)	**0.019**
Intervention type				0.934
Surgical MVr	268 (64%)	106 (63%)	162 (64%)	
Surgical MVR	131 (31%)	54 (32%)	77 (31%)	
Transcatheter MVr	21 (5.0%)	8 (4.8%)	13 (5.2%)	
Echocardiography variables				
MR grade				**0.024**
Moderate	57 (14%)	25 (15%)	32 (13%)	
Moderate-to-severe	76 (18%)	40 (24%)	36 (14%)	
Severe	287 (68%)	103 (61%)	184 (73%)	
Vena contracta, cm	0.70 (0.60–0.80)	0.60 (0.55–0.80)	0.70 (0.60–0.80)	**0.003**
PISAr, cm*	1.00 (0.80–1.20)	0.85 (0.70–1.00)	1.10 (0.90–1.30)	**<0.001**
EROA, cm^2^*	0.50 (0.30–0.75)	0.35 (0.22–0.50)	0.60 (0.40–0.80)	**0.018**
RVol, mL*	72 (51–105)	51 (43–83)	88 (67–108)	**0.013**
LVEF, %	60 (53–65)	60 (55–65)	59 (52–65)	0.234
LVEF ≤60%	241 (57%)	94 (56%)	147 (58%)	0.629
LVESDi, mm/m^2^	19.5 (17.0–22.2)	20.0 (17.6–22.4)	19.5 (16.7–22.0)	0.297
LVESDi ≥20 mm/m^2^	186 (44%)	77 (46%)	109 (43%)	0.602
LA volume index, mL/m^2^	70 (55–88)	73 (55–88)	68 (53–88)	0.120
LA volume index ≥60 mL/m^2^	256 (61%)	111 (66%)	145 (58%)	0.079
PASP, mm Hg	40 (31–53)	40 (30–50)	39 (31, 55)	0.263
PASP >50 mm Hg	105 (25%)	38 (23%)	67 (27%)	0.358
≥Moderate TR	98 (24%)	48 (29%)	50 (20%)	**0.047**
Mitral stenosis				0.543
Mild	10 (2.4%)	6 (3.6%)	4 (1.6%)	
Moderate	10 (2.4%)	3 (1.8%)	7 (2.8%)	
Severe	4 (1.0%)	1 (0.6%)	3 (1.2%)	

Significant p values are shown in bold.

* PISAr, EROA and RVol available in 96, 58 and 53 patients, respectively.

AF, atrial fibrillation; BMI, body mass index; CABG, coronary artery bypass graft; COPD, chronic obstructive pulmonary disease; CVA, cerebrovascular accident; EROA, effective regurgitant orifice area; LVEF, left ventricular ejection fraction; LVESDi, left ventricular end-systolic diameter indexed; MI, myocardial infarction; MVr, mitral valve repair; MVR, mitral valve replacement; PASP, pulmonary artery systolic pressure; PISA, proximal isovelocity surface area; RVol, regurgitant volume; SAVR, surgical aortic valve replacement; TR, tricuspid regurgitation; TVr, tricuspid valve repair.

On TTE, females had significantly lower VC (0.60 (0.55–0.80) vs 0.70 (0.60–0.80) cm, p=0.003) (figure 1), PISAr (0.85 (0.70–1.00) vs 1.10 (0.90–1.30) cm, n=96, p<0.001), EROA (0.35 (0.22–0.50) vs 0.60 (0.40–0.80) cm^2^, n=58, p=0.018), RVol (51 (43–83) vs 88 (67–108) mL, n=53, p=0.013) and MR grade (II/III/IV: 25 (15%)/40 (24%)/103 (61%) vs 32 (13%)/36 (14%)/184 (73%), p=0.024). There were no significant sex differences in LA volume index, LVEF, indexed LV end-systolic diameter or pulmonary pressures, although females had higher grades of TR (p=0.047). No significant sex differences were seen in guideline-directed criteria for intervention (LVEF ≤60%, p=0.629; LVESDi ≥20 mm/m^2^, p=0.602; LA volume index ≥60 mL/m^2^, p=0.079; PASP >50 mm Hg, p=0.358). There were no significant sex differences in procedural success (≤mild residual MR: 163 (97.0%) vs 247 (98.0%), p=0.327).

**Figure 1:**
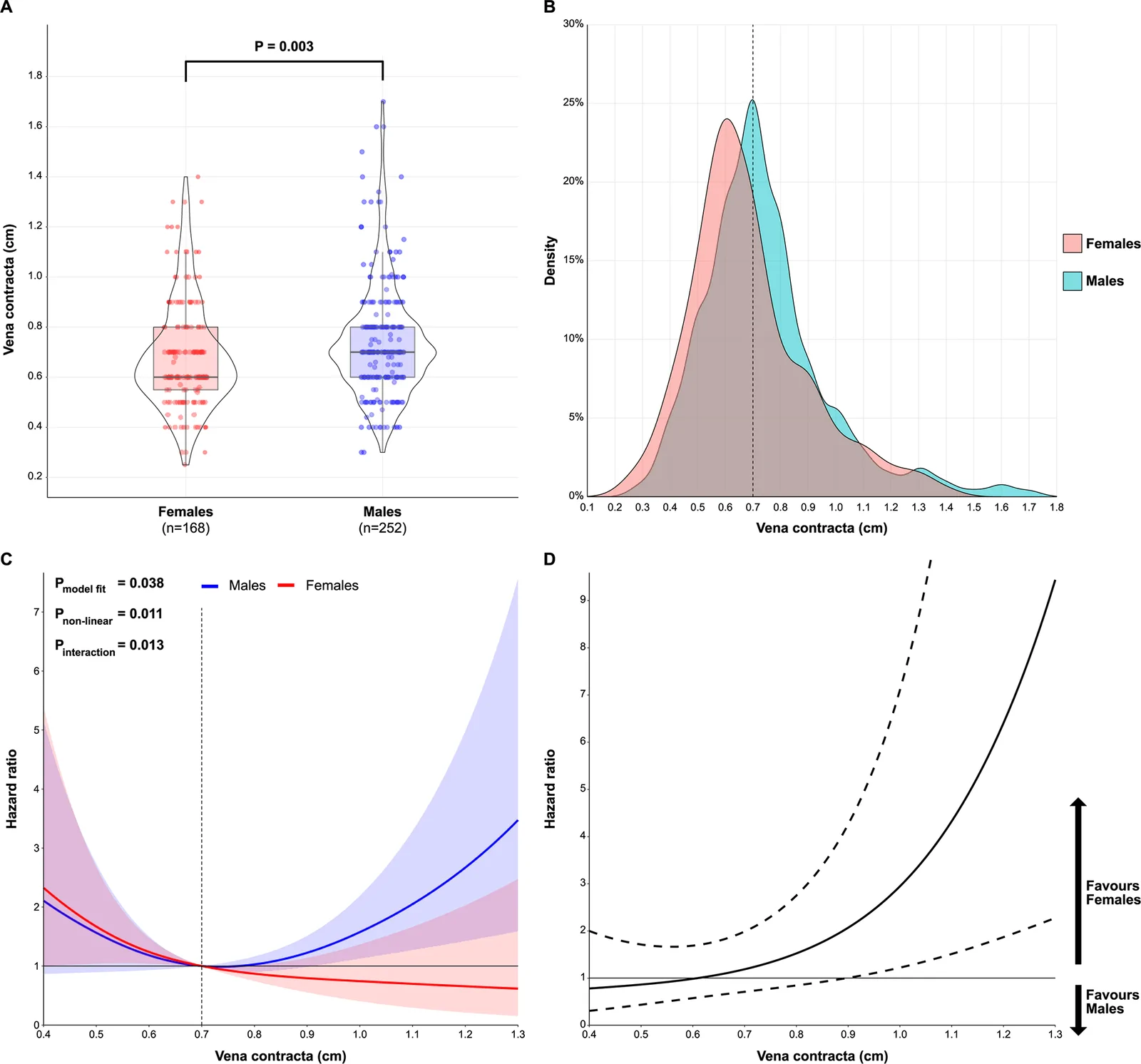
Differences in mitral regurgitation severity and prognosis according to sex. Distribution of vena contracta according to sex (**A, B**). Multivariable Cox regression analysis (**C**) demonstrated a significant interaction with sex (**D**). At higher vena contracta values, males had greater excess long-term all-cause mortality compared with females. Models adjusted for age, atrial fibrillation, left atrial volume index, left ventricular end-systolic diameter index, left ventricular ejection fraction, tricuspid regurgitation grade, and pulmonary artery systolic pressure.

Over 5.8 (2.7–9.3) years of follow-up, 105 (25.0%) patients died, with no significant sex difference (p=0.814). After adjustment, VC showed a significant non-linear relationship with all-cause mortality (figure 1) and a significant interaction according to sex (P_interaction_=0.013). Above a VC of ~0.9 cm, mortality associated with increasing VC was higher in males compared with females. In ROC curve analysis (table 2), VC had similar discriminatory ability in both sexes (p=0.768), although the optimum cut-off was lower in females than in males (females: 0.67 cm vs males: 0.74 cm).

**Table 2:** Receiver operating characteristic curve analysis according to sex

Parameter	Females	Males
AUC	0.570	0.550
Optimum vena contracta cut-off, cm	0.67	0.74
Sensitivity	64.3%	49.2%
Specificity	52.4%	62.4%

No significant difference in AUC between males and females using DeLong’s test (p=0.768).

AUC, area under the receiver operating characteristic curve.

## Discussion

In this study, we have demonstrated significant differences in TTE-derived measures of MR severity between males and females. Our key findings can be summarised as follows: (1) At the time of PMR intervention, females have significantly lower values of TTE-measured VC, PISAr, EROA and RVol compared with males; (2) sex-related differences in long-term all-cause mortality were moderated by VC; (3) sex-differences in risk calibration showed that the optimum threshold of VC for predicting all-cause mortality was lower in females compared with males.

The independent relationship between higher VC and relative excess male mortality may be explained in the context of our study population capturing patients at the time of PMR intervention. As females with PMR may be under-referred, those with very severe PMR who are selected for intervention may represent a subgroup with favourable physiological compensation. In contrast, males may be more uniformly referred for timely intervention, with a net appearance that females with very severe PMR have superior survival compared with their male counterparts. Indeed, among medically managed patients with PMR, increasing MR severity is associated with a larger increase in mortality in females relative to males,^7^ while longitudinal studies have shown that female patients with severe PMR are less likely to undergo cardiac surgery.^8^

In the context of existing literature, Altes *et al* demonstrated that optimal TTE cut-off values of EROA and RVol for the prediction of LV dilation were consistently lower in women than in men.^5^ Similarly, we also found that VC, PISAr, EROA and RVol were significantly lower in female patients compared with males. This may be a consequence of the fact that absolute regurgitant measures scale with ventricular size, and females have smaller ventricular and stroke volumes compared with males. Females also more frequently present with bileaflet prolapse compared with males, in which EROA and PISA-derived measurements can underestimate severity.^5 8^

The present study builds on these observations by directly linking MR severity, quantified by VC, to sex-related differences in long-term outcomes and demonstrating that a lower VC cut-off in females yields superior discriminative ability. Taken together, these findings suggest that sex-specific assessment of MR severity with distinct thresholds may enable earlier referral of all females, reducing excess mortality in females with extremely severe MR who may not undergo timely intervention. Thus, evaluating the use of lower absolute thresholds of MR severity to trigger intervention in female patients with PMR represents an important area of future research.

The retrospective single-centre nature of this study has inherent limitations and should be considered hypothesis-generating. In particular, preoperative cardiac symptoms were not available in this cohort and we did not have data on heart failure admissions. Moreover, PMR encompasses heterogeneous pathophysiological entities, including rheumatic heart disease, which were not specifically recorded in this cohort. Thus, differences in symptomatology and PMR aetiology should be considered as potential contributors to variability in sex-related differences in outcomes. Furthermore, VC was the only available quantitative measure of MR severity in all patients and the small number of patients with PISAr, EROA and RVol precluded performing further statistical analyses. VC is relatively less quantifiable than PISAr, EROA and RVol and may be affected by systolic/diastolic changes in regurgitant flow, although it has previously been shown to have good agreement with EROA and RVol, alongside angiographically graded MR severity.^9–12^ Thus, these findings cannot be assumed to apply to all TTE measures of MR severity. Future studies should therefore seek to replicate our findings across multiple parameters including EROA, RVol and PISAr. The inclusion of multiple types of interventions and concomitant procedures introduces heterogeneity, but this reflects real-world practice and so strengthens translatability.

## Conclusion

The present study demonstrates significant sex differences in TTE-measured MR severity and subsequent prognosis following intervention for PMR, which may merit individualised thresholds. Larger prospective studies are warranted to further characterise sex-related differences in PMR assessment using a multiparametric severity assessment including PISAr, EROA and RVol, and to determine whether using lower MR severity thresholds to trigger intervention in female patients improves long-term outcomes.

## Data Availability

Data are available upon reasonable request.
